# Pear-Shaped Lesion of the Fossa of Rosenmüller

**DOI:** 10.5334/jbr-btr.921

**Published:** 2016-07-18

**Authors:** B. Peters, K. De Cuyper, F. M. Vanhoenacker

**Affiliations:** 1 Dept. of Radiology, AZ Sint-Maarten, Duffel-Mechelen, Rooienberg 25, B-2570 Duffel, Belgium; 2 Department of Radiology, Antwerp University Hospital, University of Antwerp, Edegem, Belgium; 3 Faculty of Medicine and Health Sciences, University of Ghent, Belgium

**Keywords:** Retention cyst, MRI, CT, fossa of Rosenmüller, T2-WI, Benign lesion

## Case

**Figure 1:**
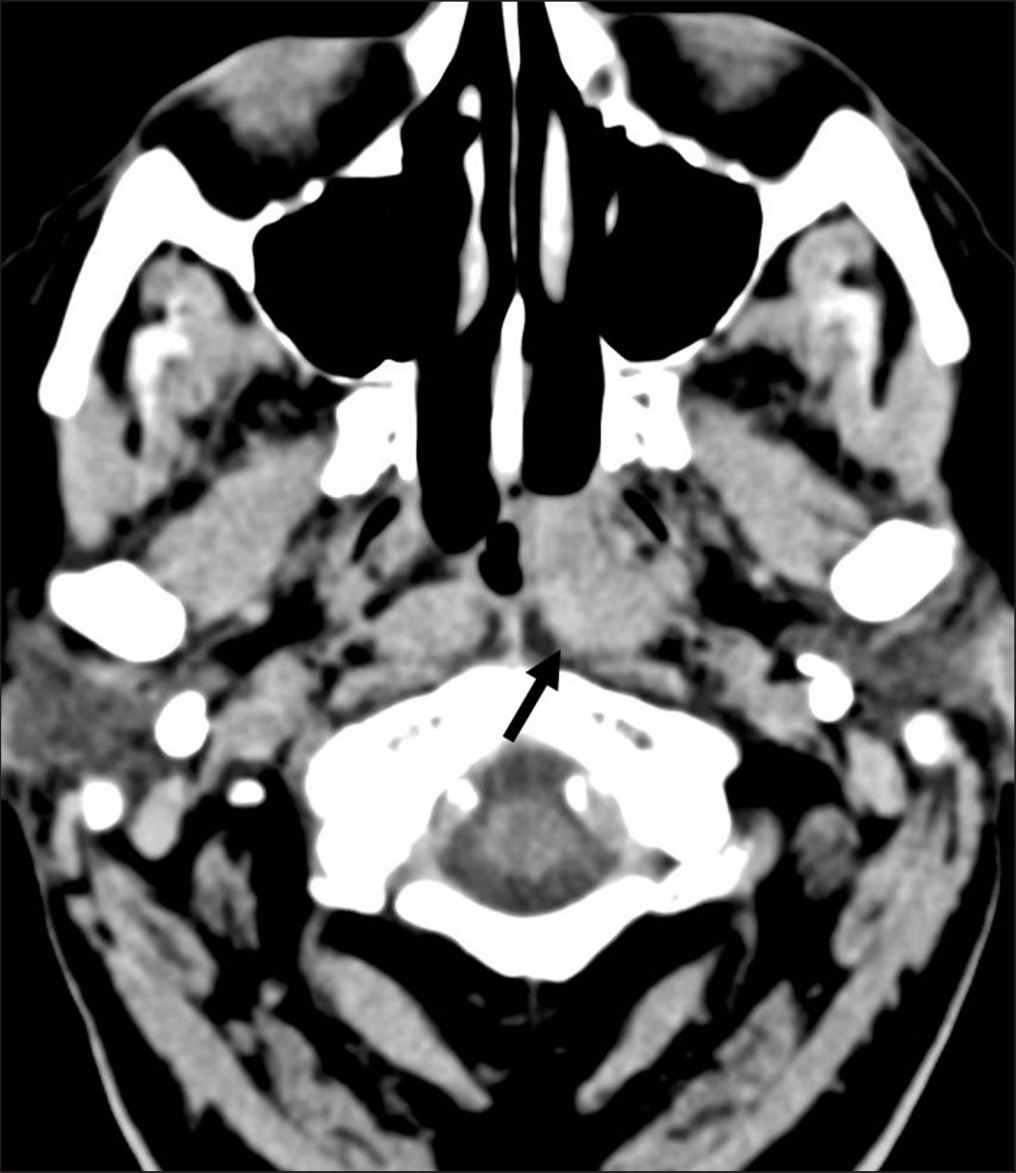


A 72-year-old man was admitted with persistent temporal headache. Further clinical history, physical examination, and laboratory tests were unremarkable. Nonenhanced computed tomography (CT) of the head (Figure 1, black arrow) revealed a lesion with nonspecific attenuation in the left fossa of Rosenmüller. For further characterization, magnetic resonance imaging (MR) was performed.

**Figure 2:**
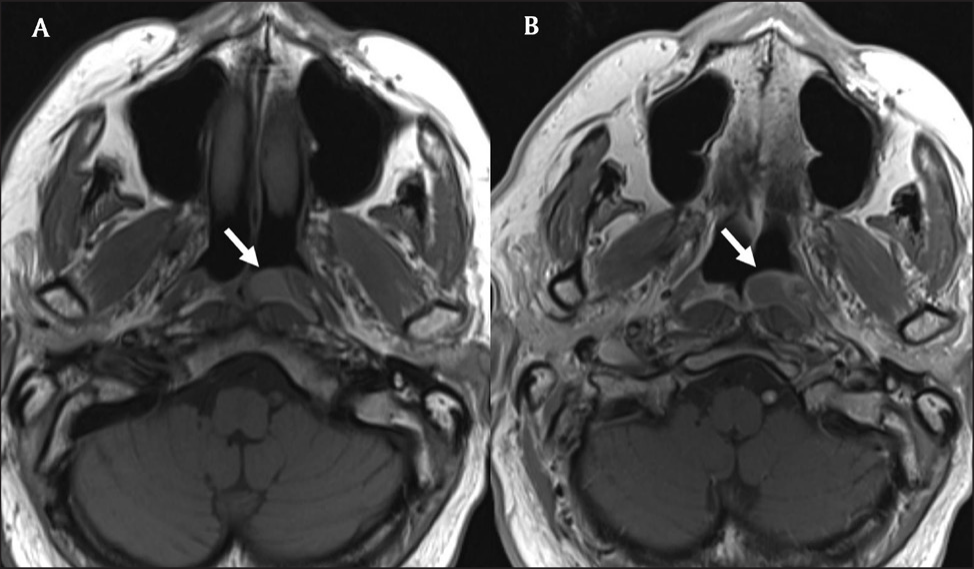


**Figure 3:**
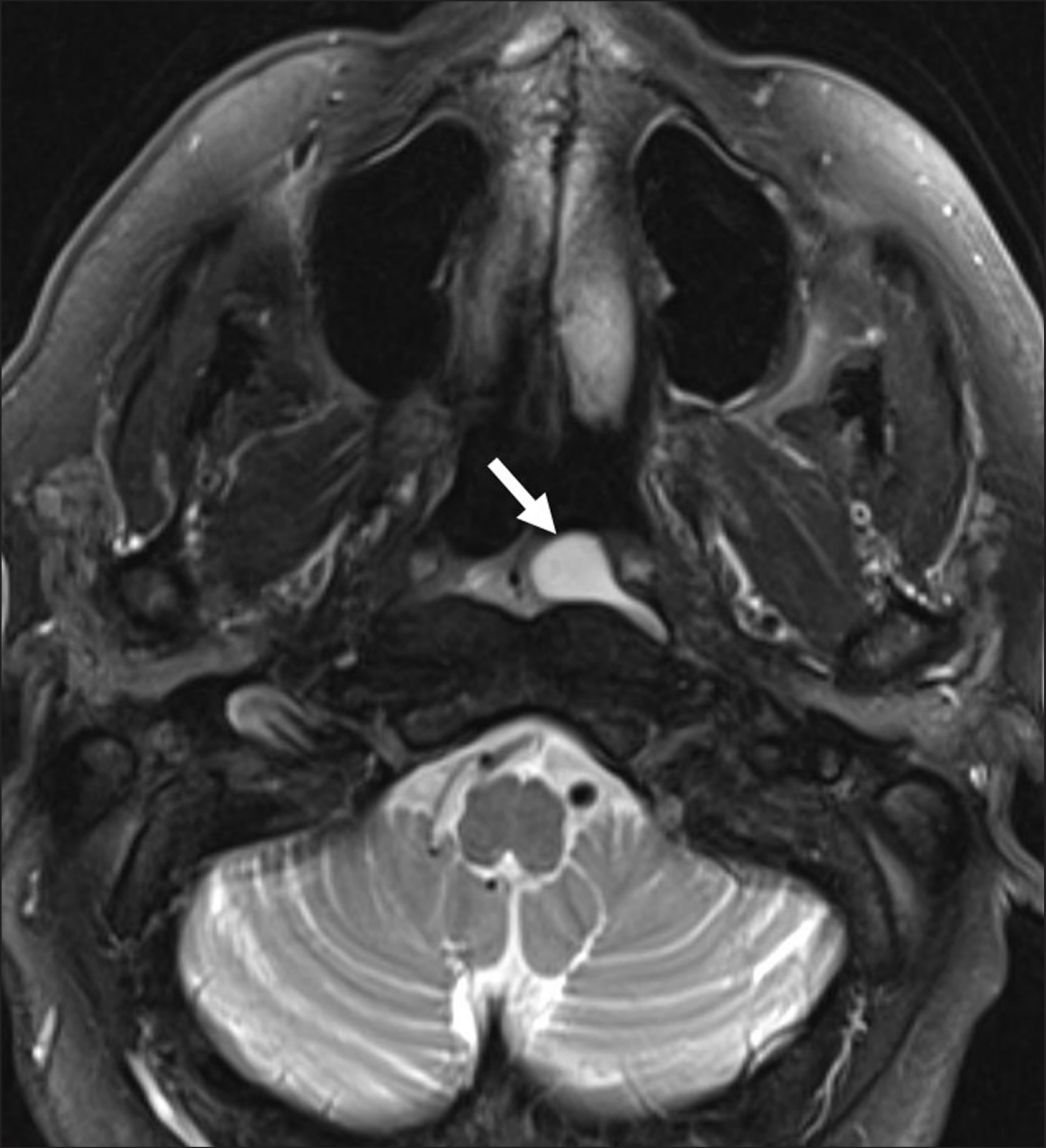


The lesion was pear-shaped and well circumscribed. The lesion was slightly hyperintense on T1-weighted images (WI) (Figure 2A, white arrow) and hyperintense on fat-suppressed T2-WI (Figure 3, white arrow). There was subtle peripheral enhancement after administration of gadolinium contrast (Figure 2B, white arrow). The diagnosis of a retention cyst of Rosenmüller’s fossa was made. As the patient was asymptomatic, watchful waiting was recommended.

## Comment

A cyst of the fossa of Rosenmüller is a benign retention cyst (RC) of the nasopharyngeal mucosa or adenoids, arising from dilated mucus glands in the lamina propria or deep layers of the pharyngeal wall secondary to accumulated secretions and chronic inflammation. RC of the oropharynx are far less common than those located at the nasopharynx.

Sekiya et al. found RC in the pharynx as an incidental finding in 10 per cent of patients in a retrospective analysis of 3,000 MRIs of the brain. RC is usually smaller than 1 cm and asymptomatic, but cysts larger than 1 cm may cause dysphagia, respiratory symptoms, rhinosinusitis, and middle ear effusion and rarely become ulcerated or secondarily infected.

RC usually appears as a cystic lesion of the pharyngeal mucosa on nonenhanced CT, without extension in the surrounding structures. Cysts with a proteinaceous content may appear slightly hyperdense, as in our case. There is no significant enhancement on contrast-enhanced CT. On T1-WI, it typically appears isointense to muscle. Often RC are slightly hyperintense, related to a higher protein content. After contrast administration, there is no or only minor peripheral rim enhancement of the wall. The preferred MR sequence is a T2-WI on which the lesion shows a homogeneously hyperintense signal [1]. There is a distinct plane between the RC and the surrounding structures. Multilocularity or intralesional septation may occur.

The differential diagnosis with an atypical localization of a second branchial cleft cyst should be made. These patients are usually symptomatic and present in early adulthood due to superimposed infection. Although the signal intensity and contrast-enhancement pattern may be similar to that of an RC, a branchial cleft cyst is typically located laterally in the neck region and has a more rounded shape. Location in the nasopharynx is very rare. In most cases, RC is a “do not touch lesion”. Rarely surgical resection is indicated, if symptomatic.

In conclusion, RC is a relatively common benign lesion of the nasopharynx often visible on routine imaging of the head or cervical spine. Rosenmüller’s fossa is a site of predilection. It is important not to misinterpret RC as a malignant tumor.
